# An Artificial Intelligent System for Prostate Cancer Diagnosis in Whole Slide Images

**DOI:** 10.1007/s10916-024-02118-3

**Published:** 2024-10-28

**Authors:** Sajib Saha, Janardhan Vignarajan, Adam Flesch, Patrik Jelinko, Petra Gorog, Eniko Szep, Csaba Toth, Peter Gombas, Tibor Schvarcz, Orsolya Mihaly, Marianna Kapin, Alexandra Zub, Levente Kuthi, Laszlo Tiszlavicz, Tibor Glasz, Shaun Frost

**Affiliations:** 1 Australian e-Health Research Centre, CSIRO, Kensington, Australia; 2 AI4Path (Prosperitree Pty Ltd), Roseville, Australia; 3 Markusovszky University Teaching Hospital, Szombathely, Hungary; 4 St. Barbara County Hospital, Tatabanya, Hungary; 5 Josa Andras Teaching Hospital, Nyiregyhaza, Hungary; 6 Department of Pathology, Albert Szent-Györgyi Medical School, University of Szeged, Szeged, Hungary

**Keywords:** Deep learning, Prostate cancer, Cancer detection, Computer-assisted diagnosis, Whole slide imaging, WSI, Convolutional neural network, CNN

## Abstract

In recent years a significant demand to develop computer-assisted diagnostic tools to assess prostate cancer using whole slide images has been observed. In this study we develop and validate a machine learning system for cancer assessment, inclusive of detection of perineural invasion and measurement of cancer portion to meet clinical reporting needs. The system analyses the whole slide image in three consecutive stages: tissue detection, classification, and slide level analysis. The whole slide image is divided into smaller regions (patches). The tissue detection stage relies upon traditional machine learning to identify WSI patches containing tissue, which are then further assessed at the classification stage where deep learning algorithms are employed to detect and classify cancer tissue. At the slide level analysis stage, entire slide level information is generated by aggregating all the patch level information of the slide. A total of 2340 haematoxylin and eosin stained slides were used to train and validate the system. A medical team consisting of 11 board certified pathologists with prostatic pathology subspeciality competences working independently in 4 different medical centres performed the annotations. Pixel-level annotation based on an agreed set of 10 annotation terms, determined based on medical relevance and prevalence, was created by the team. The system achieved an accuracy of 99.53% in tissue detection, with sensitivity and specificity respectively of 99.78% and 99.12%. The system achieved an accuracy of 92.80% in classifying tissue terms, with sensitivity and specificity respectively 92.61% and 99.25%, when 5x magnification level was used. For 10x magnification, these values were respectively 91.04%, 90.49%, and 99.07%. For 20x magnification they were 84.71%, 83.95%, 90.13%.

## Introduction

Prostate cancer is the second most common form of cancer in men [1], with more than 1 million new diagnosed cases worldwide every year [2]. Diagnosis of prostate cancer typically involves histopathological assessment of biopsy tissue. Microscopic examination of tissue sections stained with haematoxylin and eosin (H&E) are the most commonly used rendering techniques for tissue diagnosis by pathology laboratories [3]. Manual assessment of biopsy tissue is time consuming and requires specific expertise, for which there is a growing shortage worldwide. In recent years there has been an overall increase in cancer incidence and a dramatic increase in the number of biopsies reviewed per case [4]. For these reasons, there is an emerging need to develop automated screening and decision support tools for prostate cancer assessment. Research into artificial intelligence (AI)-based tools applied to digital microscope images of biopsies is progressing to meet this need.

Over the last decade, the technological and regulatory advances of digital whole slide imaging (WSI) have accelerated its adaptation in histopathological assessment [5]. In recent years with increasingly more pathology departments having access to commercial digital imaging platforms for diagnostic work, there is growing interest to develop AI tools to support pathologists. A few computational pathology algorithms have already been developed for automated disease diagnosis, prognosis and prediction of treatment response using WSI [6]. In the context of prostate cancer, these approaches can be broadly divided into two groups – 1) traditional rule-based methods, and 2) deep learning-based methods.

In traditional rule-based methods, human crafted features are extracted from the images. Classifiers such as support vector machine (SVM) or random forest (RF) are then applied to classify prostate tissue into stroma, benign/normal epithelium, and cancer. Prior to feature extraction, image pre-processing is typically performed to analyze textural complexity, edge and segmentation information. Several methods in this category have been published [6–11].

Deep learning methods (a type of machine learning algorithm) employ convolutional neural networks (CNNs) to classify WSI patches into benign / malignant or into Gleason grades [12–21]. A majority of the published methods adopted CNNs that were originally developed to solve other computer vision problems and applied transfer learning to fine-tune them in the context. For example, [22] retained and fine-tuned YOLO-v3, a general-purpose object detection algorithm, for automated diagnosis and grading of prostate cancer. Only a few methods considered developing novel CNNs in the context or significantly altering the conventional architecture specific to the context. [16] method proposed a deep learning system named DeepDx. The system comprises two steps: patch-level segmentation and slide-level evaluation. In the first step, the input WSI is split into patches of size 704 × 704 pixels. Each of the patches are then segmented with respect to five categories: non-cancerous; Gleason patterns 3, 4, and 5; and IDC-P. In the second step, the patch results in a slide are aggregated into a single heatmap. Another example is Poojitha et al.’s method [20]. The architecture combined CNN, recurrent neural network and fine-tuned VGG net. [23] proposed a weakly supervised deep learning model named MIL-GCN. MIL-GCN employs a self-supervised pretrained encoder to extract features, and which are then are aggregated using GCN. Finally, the labels are predicted with an attention-based MIL. [24] developed an automated tumour assessment and classification model named ATARI. They also trained ResNet101, a general-purpose CNN, for the same task, and performed a comparative evaluation among them. For a detailed review about the available methods in the context, readers are directed to the paper of [24].

Deep learning-based models in general have been found to outperform traditional rule-based methods [2] and have shown promising results to assess prostate cancer using histopathology images. However, most of these methods are limited to cancer detection and Gleason grading only, and do not provide insight about perineural invasion and/or cancer portion to meet clinal reporting needs. This paper aims to bridge this gap.

In line with the recent trends [2], we have grouped together some Gleason grades based on clinical significance, thus increasing the available data for each classification while retaining clinical relevance.

Specific contributions to this study include:

1. Development and validation of a multi-step machine learning system. The system not only performs cancer detection but also detects perineural invasion and measures cancer portion to meet clinical reporting needs.
2. Extensive validation and testing on a multi-centre dataset of 2,340 WSI samples, which are also collected as part of this study.
3. Use of annotations relying upon inputs from 11 board certified pathologists with prostatic pathology subspeciality competences, that are also generated as part of this study.

## Materials and Methods

### Data Preparation

In compliance with the project concept, a medical team consisting of 11 board certified pathologists with prostatic pathology subspeciality competences working independently in 4 different medical centres was organized aiming to create prostatic adenocarcinoma annotations on anonymized whole slide images (WSI) of prostate core biopsy specimens by means of a widely used, open-source annotation platform (Cytomine, Liege, Belgium). Our study design aimed therefore to emphasize human quality diagnostic work, generally a bottleneck in similar studies [13, 17, 21]. Cooperation of the 4 medical centres and the project management as well as the project concept had previously been consented by local bioethical authorities. A pool of altogether 2,340 WSI was created by the medical team via initial computer searching in the archives of the 4 participating medical centres for prostatic core biopsy cases previously diagnosed as cancer positive (1 biopsy per patient. Hematoxylin and eosin (HE stained glass slide specimens of each archived case were quality controlled as well for slide physical properties as for cancer diagnosis, then scanned at 20 × magnification by appropriate slide scanner machines (1 WSI per case,3DHistech, Budapest, Hungary. WSI-s were anonymously uploaded to our project dedicated server wherefrom the Cytomine annotation platform could be operated to grant access for each medical team member to personally dedicated and password-protected cases. An initial pixel-level annotation round of all cases was based on a previously agreed set of 49 annotation terms, similar to that described by others [2] This was in the long run further focused to a subset of 10 terms that finally proved to be both medically relevant and prevalent enough to constitute the medical data package worth of transferring for IT-processing (Table 1). The WSI population was split into a training data set (1,904 WSI) and a hold-out data set (436 WSI), and on both data set the medical team developed annotations for AI model training and then subsequently for model performance testing. To ensure that the annotations in the validation set were accurate (acting as the source of truth), each annotation in the validation set had been reviewed by a second medical team member. If first- and second-look opinions proved to be in agreement, consensus was declared and annotation judgement was accepted, whereas upon disagreement, a third reviewer’s decision had been sought. In case the three experts had got to three different results, the annotation was excluded for lack of consensus.

**Table 1:** Focused List of Annotation Terms

Classified area type	
All classified tumor components	
	Gleason pattern 3 tumor structures (glands)
	Gleason pattern 4 tumor structures (confluent glands)
	Gleason pattern 4 tumor structures (cribriform glands)
	Gleason pattern 5 tumor structures (glands)
	Perineural tumor invasion
All classified non-tumor components	
	Atrophic prostate gland
	Basal cell hyperplasia
	Chronic inflammation area
	Prostatic gland, LG PIN, non-basal hyperplasia (glands)
	Seminal vesicle epithelium

#### Patch Generation

**Fig. 1:**
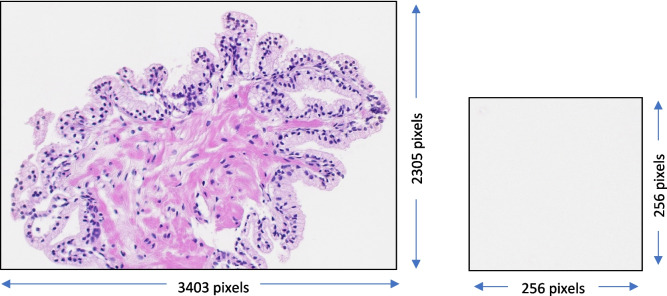
**a** An example ROI at 10 × magnification level that has big enough non-tissue regions to be independently considered as a patch. **b** an example non-tissue patch generated by cropping the ROI shown in a

Experts drew polygons, using Cytomine (https://cytomine.com/) outlining each of the 10 tissue types present in the WSI. For each of the polygons, using an in-house developed software program we created a bounding box and cropped and saved that region as independent image. Images were saved in.png format and no compression was applied while saving. For simplicity we will call these images as region of interests (ROIs). From each ROI we created about *Z*_*i*_ (${\forall }_{i}\in \text{total number of ROIs}$) number of 256 × 256 patches by cropping. Here, *Z*_*i*_ is the integer equivalent of${N}_{i}=\frac{{W}_{{ROI}_{i}}\times {H}_{{ROI}_{i}}}{256\times 256}$, where ${W}_{{ROI}_{i}}$ and ${H}_{{ROI}_{i}}$ are respectively the width and height of the ROI in consideration. When 0.5 ≤ ${N}_{i}<1$, we created a patch of size 256 × 256 by padding required number of pixels. The color values of the padded pixels were set to (240, 240, 240), which is the mean intensity of the background, determined experimentally. When ${N}_{i}<0.5$ the associated ROI has been ignored and no patch was generated out of this ROI. Each of the generated patches were independently checked whether it was truly tissue or not. Figure 1 shows a particular example where ROI had non-tissue regions inside and which was big enough (i.e.,$\ge 256\times 256$) to be independently considered as a patch.

To ensure fair training of the CNNs in this work, in line with other works in the literature, we decided to use a similar number of patches for each of the 10 terms, and thus adjusted *Z*_*i*_ accordingly.

Patches were generated independently for each of the magnification levels—5x, 10x and 20x, however, the patch size for each of the magnification level were kept same, and this had been inspired by the work of [2]. Likewise, we adopted the [2] patch size as $256\times 256$. Table 2 details the number of patches used for training and validation of the CNNs.

**Table 2:** Total number of patches in each term and magnification level

Term ID	Term details	No. of patches		
		5x	10x	20x
1	Benign: basal cell hyperplasia	3,579	5,256	5,859
2	Benign: atrophic prostate gland	4,262	5,009	6,213
3	Benign: prostatic gland, LG PIN, non-basal hyperplasia (gland)	4,516	5,022	6,383
4	Benign: seminal vesicle epithelium	4,219	5,160	6,612
5	Benign: chronic inflammation (area)	3,470	4,952	6,248
6	Cc: Gleason3 tumor structure (gland)	1,853	4,975	6,011
7	Cc: Gleason4 tumor structure (cribriform gland)	3,627	5,268	6,461
8	Cc: Perineural tumor invasion	3,035	5,100	6,392
9	Cc: Gleason5 tumor structure	4,684	4,996	6,269
10	Cc: Gleason4 tumor structure (counfluent gland)	5,083	5,195	6,022

We randomly split the dataset into—training set (90%) and—validation set (10%). An additional independent test set of size approximately 19% of the full data was used for the final testing of classification performance.

### Proposed System

**Fig. 2:**
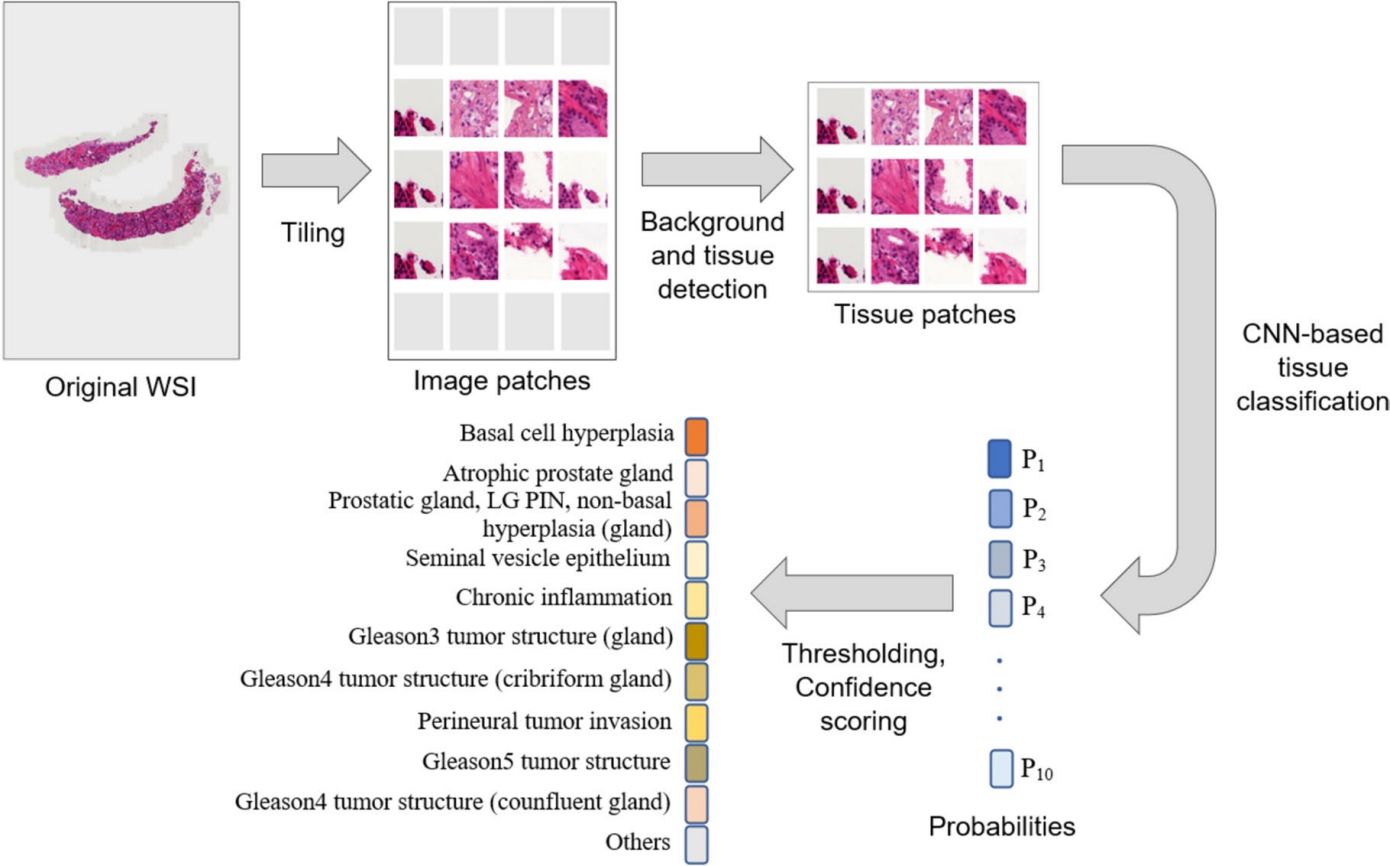
The process of classifying a whole slide image (WSI) with the proposed system. The input WSI is first divided into square patches of predefined sizes. These patches then sequentially pass-through rule-based machine learning and deep learning-based methods and are classified

The system analyses the whole slide image (WSI) in three consecutive steps that include tissue detection, classification, and slide level analysis. Given WSI as input the system first divides the image into multiple patches of size 256 × 256. This process is called tiling. A supervised method [25] that relies on traditional rule-base machine learning (Jimenez-del-Toro et al*.* 2017) has been developed to identify tissue and non-tissue (or background) patches. Deep convolutional neural networks (CNNs) [26] have been trained to classify each tissue patch into 10 predefined classes (Table 1). These classes were chosen based on their clinical significance in prostate biopsies and/or their distinct morphologic appearances. Transfer learning [27] has been used in training the CNNs. From the list of CNNs that were available for further development and training we choose to experiment with Inception-v3 [28], ResNet50 [29] and VGG16 [27], who are widely used and best performing CNNs in several computer vision tasks. Ten heatmaps have been generated by feeding all of the tissue patches generated from a WSI to the CNN, where each heatmap represents the probabilities of one class along the entire slide. From the heatmaps a set of scores per slide that quantify the likelihood of cancer has been computed. Figure 2 shows the overall process of the proposed system.

The system was implemented in Python, version: 3.8.12 (http://www.python.org). Keras, version: 2.4.3(https://keras.io/) with Tensorflow, version: tensorflow-gpu-2.3.0 (https://www.tensorflow.org/), was used for the development of the CNN. Openslide, version: openslide-python-1.1.2 (https://openslide.org/) was used to read WSI.

The training, validation and testing was performed on a Dell Precision 5820 Tower Workstation, which had an Intel Xeon 3.60 GHz CPU installed. The workstation was equipped with 64 GB RAM, and an NVIDIA GeForce RTX 2080Ti GPU (Dell Inc., Round Rock, TX, USA).

#### Tissue Detection

The tissue detection step runs on a patch of size 256 × 256 and classifies each patch as tissue or non-tissue (or background). For each of the patch a 24-dimensional(D) feature vector is computed to describe the patch. Statistical measures that include mean, standard deviation, variance, median, minimum, maximum, range, and mode [30] computed independently on each of the red, green and blue channels of the patch are computed and finally combined to form the 24-D feature vector that described the patch. A random forest (RF) classifier [31] has been trained to perform the background and tissue classification based on the feature descriptor.

While implementing the RF classifier we experimented on a list of parameters and thus ensured the classifier is optimally trained in the context. The parameters we experimented on include – the number of trees in the forest (‘n_estimators’) [32], which function to use as criterion to measure the quality of a split (‘criterion’) [33], when to stop expanding the leaves (‘max_depth’), the minimum number of samples required to be at a leaf node (‘bootstrap’) [33], and how to setup the “bootstrap” parameter [32]. We found the setup—‘n_estimators’ = 100, ‘criterion’ = ‘entropy’, ‘max_depth’ = None and, ‘bootstrap’ = True, works best in the context. More specifically, We experimented with the following set combination of hyperparameters while training the RF classifier: ‘n_estimators’: [50, 100, 200], ‘criterion’: ['gini', 'entropy'], ‘max_depth’: [None, 10, 20, 30], and ‘bootstrap’: [True, False]. To systematically evaluate all possible combinations of these hyperparameters, we utilized the GridSearchCV object in scikit-learn [33]. This tool performs an exhaustive search over the specified parameter values using cross-validation. After evaluating all combinations, the ‘grid_search.best_params_’ attribute stores the combination of hyperparameters that achieved the highest score according to the specified scoring metric (i.e., accuracy).

**Fig. 3:**
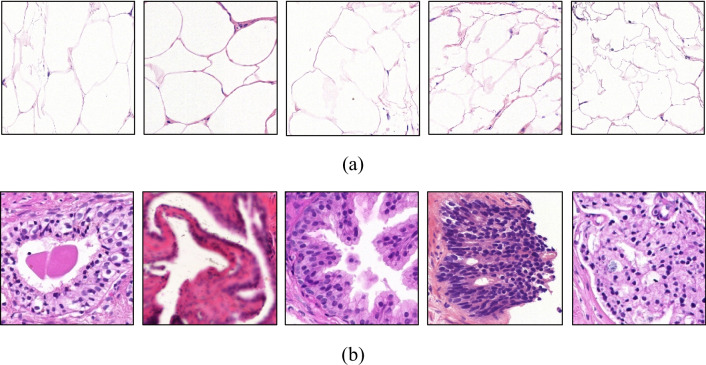
Sample (**a**) fat-tissue and (**b**) other tissue patches used to train the RF classifier

A total of 15,034 patches randomly chosen from 50 whole slide images were extracted and labelled as ‘tissue’ or ‘background’ for training and validation of the classifier. The number of patches on each category were exactly half of the total number of patches. A set of 2,255 fat-tissue patches were explicitly included in the ‘tissue’ set. This was to ensure the sensitivity of the RF classifier is high enough to detect fat tissue that may be repeatedly present at the periphery of tissue specimens. It is worth mentioning that ‘fat-tissue’ gives a very whitish hue that may easily be taken for background whiteness. Figure 3 shows some sample fat-tissue patches along with other tissue patches.

#### Tissue Classification

##### Pre-Processing and Data Augmentation

The patches were pre-processed to transform them into an appropriate input specific to the model. More specifically, patches were first converted from RGB (R = Red, G = Green, B = Blue) to BGR, then each color channel was zero-centered with respect to the ImageNet dataset [34], without scaling. The ‘preprocess_input()’ function specific to VGG16 and readily available in Keras was used for that purpose.

Data augmentation was also performed prior to training the CNNs. Data augmentation is a common practise to train deep learning models because it generates new training data that helps to prevent over fitting and increases the generalizability of the model [35]. For the data augmentation we used the Keras ‘ImageDataGenerator()’ function which generates a continuous stream flow of data to the model for the training phase, with options for all commonly used image augmentation techniques. In this work we used rotation between 0 and 360 degrees, shear in the range 10%, zoom-in and zoom-out by 10%, width shift range 10%, height shift range 10%, and horizontal flipping.

##### Training the CNN

Inspired by the work of [2], in this work we decided to experiment on 3 different magnification levels (i.e., 5x, 10x, 20x). However, in different to Pantanowitz et al*.* rather than ensembling them directly in this work we critically analyze their respective performance. Each of the CNNs are independently trained using WSI patches of 3 different magnification levels. We experimentally found (details in Appendix) VGG16 was performing at least as good as the other CNNs, specifically Inception-v3 and ResNet50 in the context, and thus we decided to persist with VGG16 throughout the study. VGG16 is simple, yet one of the best performing and most frequently used CNNs [36]. Since our target outputs (i.e., number of classes) are different than the ones from the original model, to adapt the model in our context we removed the top layer of the CNN and added a global average pooling 2D layers and finally a dense layer with 10 outputs. Softmax activation was used. Glorot uniform initializer, also known as Xavier uniform initializer [37], was used to initialize the weights of the newly added layers.

We initialized the parameters of the model using transfer learning [38]. More specifically, we used pre-trained weights downloadable from (VGG16_weights), to initialize the model parameters. These pre-trained weights were originally computed by training VGG16 on ImageNet dataset [34], which is one of biggest classification datasets till date [39]. Once initialized, we then fine-tuned the weights using our dataset. It is worth mentioning, initialization with pre-trained weights over random weights ensures fast network training with less epochs, avoids over fitting and ensures robust performance [35]. It is a promising alternative to full training and is a common practise nowadays [38]. ‘Stochastic gradient descent (SGD)’ [40] optimizer was used with learning rate 0.0001 and momentum 0.9. ‘Categorical Cross Entropy’ was used as the loss function. To train the model, we firstly trained the newly added layers for 20 epochs and then trained the entire model for another 200 epochs.

#### Slide Level Analysis

**Fig. 4:**
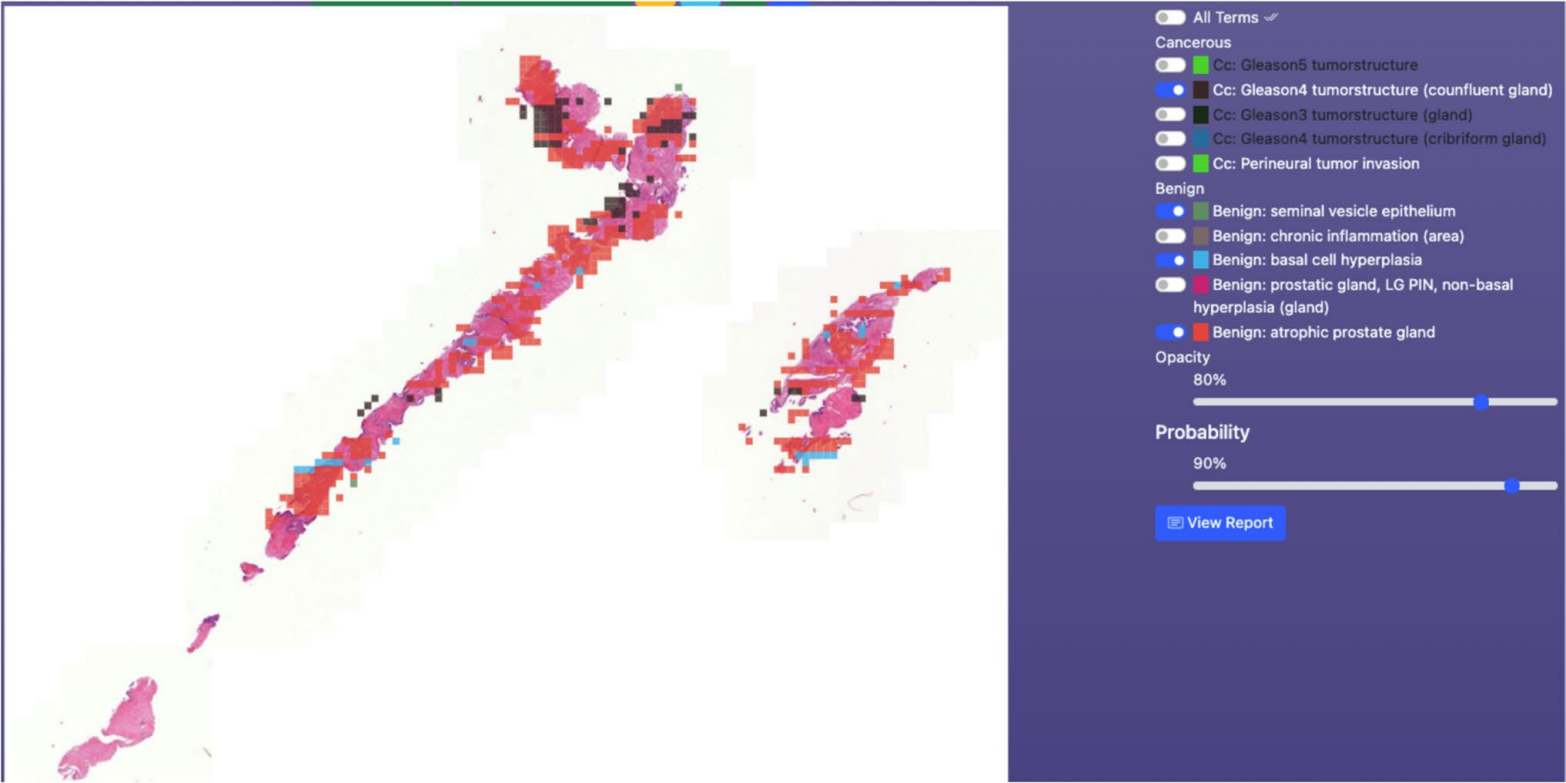
Sample heatmap produced by the system

**Fig. 5:**
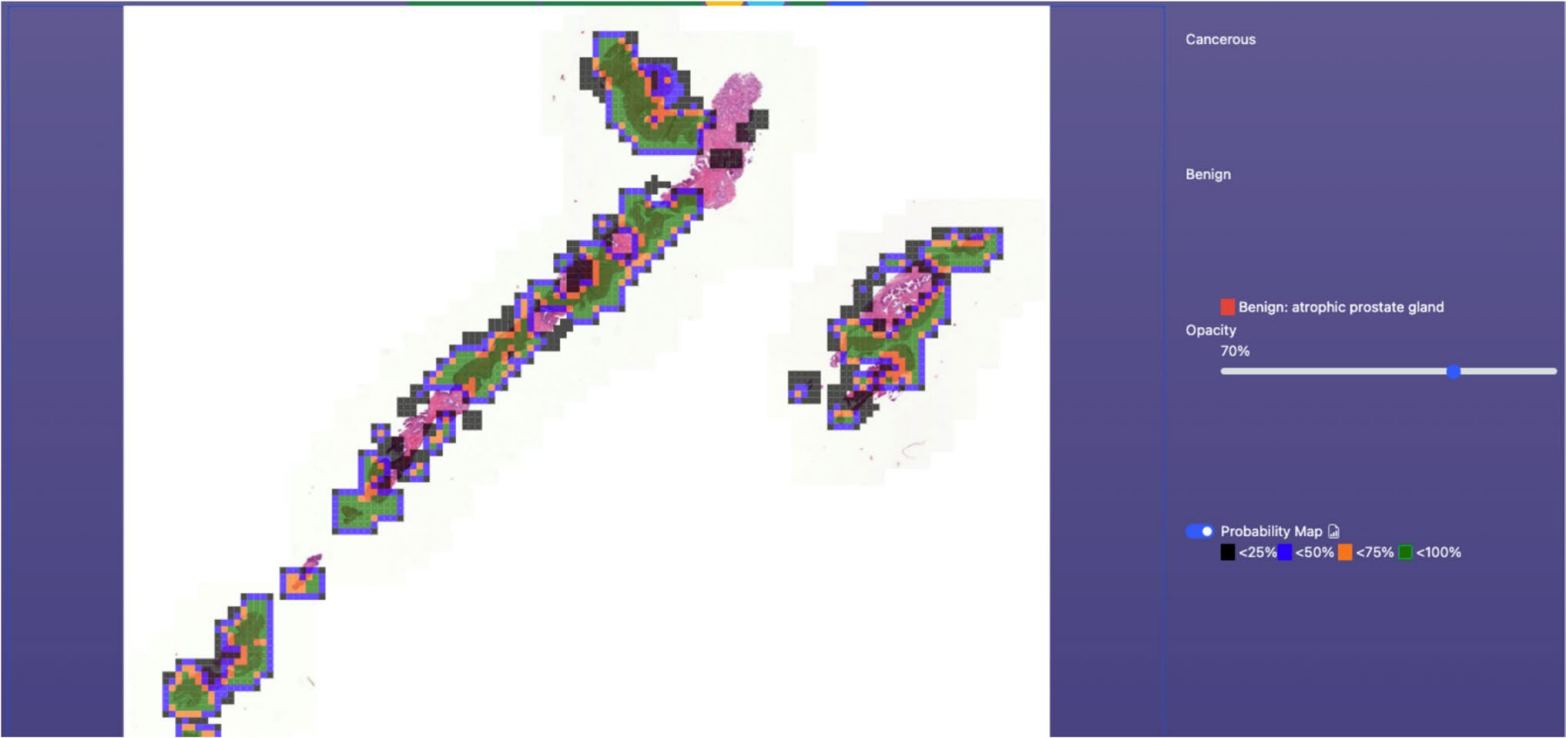
Another presentation of the heatmap produced by the system

Entire slide level information is generated by aggregating all the patch level information and mapping it back to original slide level. A total of 10 heatmaps are generated by considering the patch-wise probability of each of the 10 predefined terms. Thus, each heatmap represents the probabilities of one class along the entire slide. Figures 4 and 5 show some example heatmaps generated by the system.

**Fig. 6:**
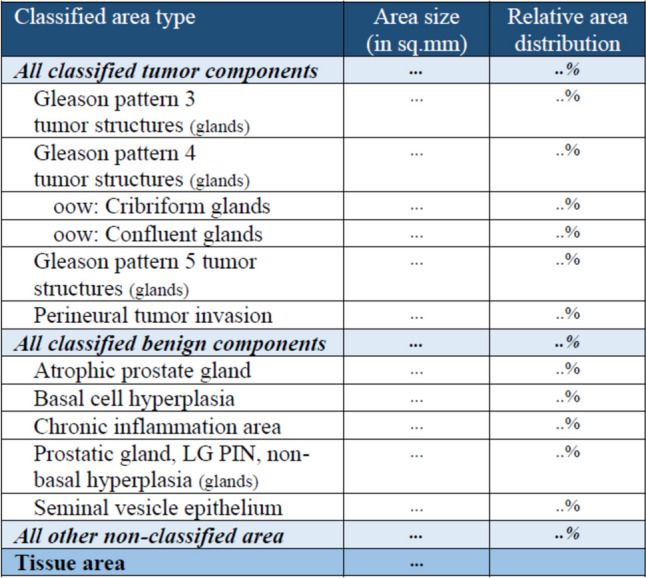
Basic area data

Each of the heatmaps is independently used to compute ‘area size’ (in sq.mm) and ‘relative area distribution’. To compute these values a threshold ${T}_{h}$ is applied to the heatmap in consideration.${T}_{h}$,{${\forall }_{h}|h\in 1:10\}$ is a user defined input (default: 0.5). Probabilities that are greater than or equal to ${T}_{h}$ are set to 1 and rest are set to 0. A value${S}_{h}$, which is summation of all the ‘1’s in heatmap *h*, is computed for each of the 10 heatmaps. To compute the ‘area size’ in sq. mm, ${S}_{h}$ is multiplied by the corresponding ‘x_resolution’ and ‘y_resolution’ that comes with the WSI file (e.g.,.ini). To compute the ‘relative area distribution’ ${S}_{h}$ is divided by the total tissue present in the WSI. Figure 6 shows a snapshot of the basic area data computed from the heatmaps.

**Fig. 7:**
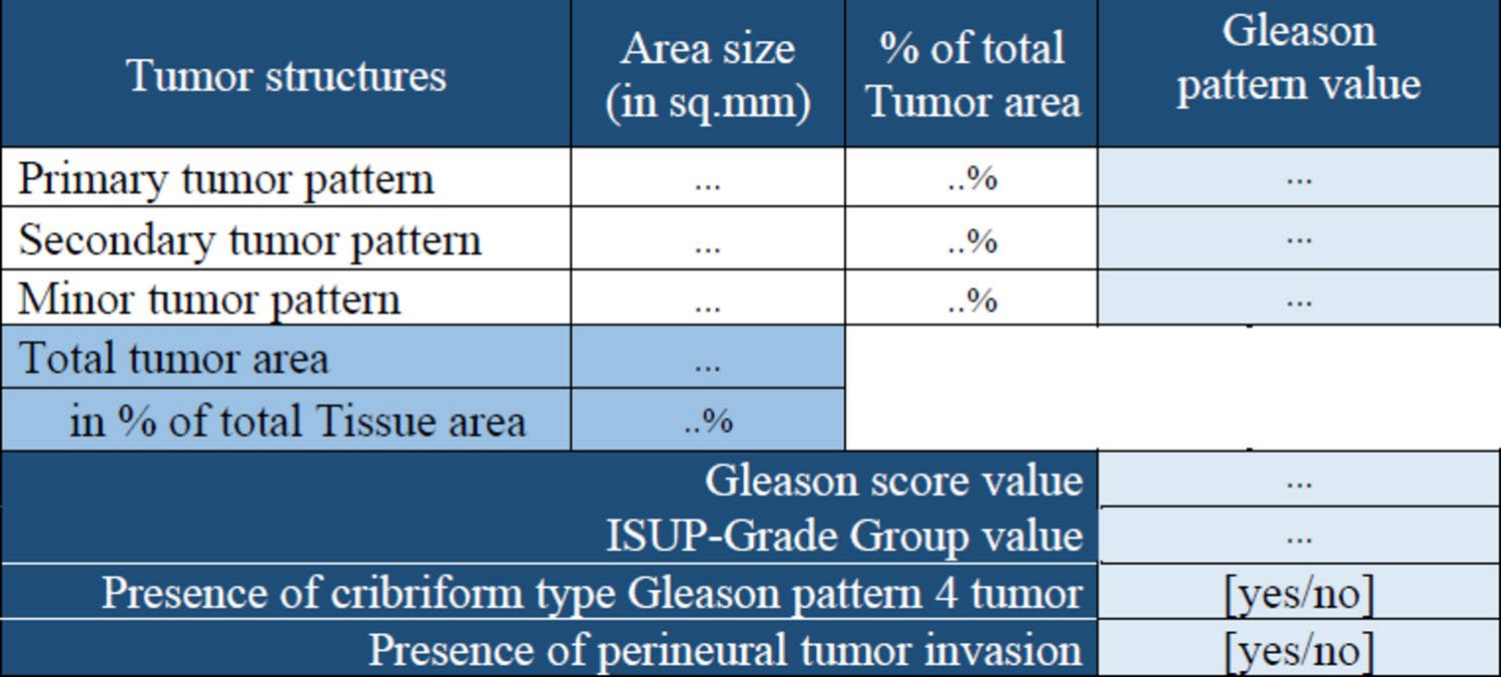
Gleason scoring data

From the basic area data more specific Gleason scoring data is computed (Fig. 7).

The system provides user the option to choose which model to run – 1) 5x, 2) 10x, 3) 20x or 4) combined, while performing cancer assessment. While combined option is chosen – 1) the strides of the three models are automatically adjusted in a way such that they will produce heatmaps of the same size, 2) the heatmaps produced by 5x, 10x and 20x are aggregated by taking the weighted average (specified by the user, default 1/3) of them.

### Evaluation Metric

The commonly used measures of evaluation that include sensitivity, specificity, accuracy, and area under the receiver operating characteristics curve (AUC) have been used throughout this study to analyse the performance of the system [41]. Sensitivity, specificity, and accuracy are mathematically defined as follows:

1 $$\text{Sensitivity}=\frac{TP}{TP+FN},$$

2 $$\text{Specificity}=\frac{TN}{TN+FP},$$

3 $$\text{Accuracy}=\frac{TP+TN}{TP+TN+FP+FN}.$$

Here *TP*, *TN*, *FP*, and *FN* represent true positive, true negative, false positive and false negative respectively. In the receiver operating characteristics curve (ROC), the sensitivity (also called true positive rate) is plotted against the false positive rate (i.e., 1-specificity) for different cut-off points of a parameter and the area under the ROC curve is then computed.

## Results

### Tissue Detection Performance

A tenfold cross-validation was performed to evaluate the performance of the classifier. The classifier achieved an overall accuracy of 99.53%. Table 3 details the mean (over tenfold) accuracy, sensitivity, and specificity, and their respective standard deviations (std.).

**Table 3:** Performance analysis of the tissue detection step

Accuracy (Std.)	Sensitivity (Std.)	Specificity (Std.)
0.9953 (0.003)	0.9978 (0.005)	0.9912 (0.005)

### Tissue Classification Performance

While training the CNN, we experimented on 10 different setups than include fine-tuning from the top—10%, 20%, 30%, 40%, 50%, 60%, 70%, 80%, 90% and 100% of the available layers, and thus identified the best setup in the context. For a CNN with *N* layers, if ${\alpha }_{i}$ is the learning rate of the *i*-th layer in the network, *p*% fine-tuning means for all the layers, except the top *L* layers, we set ${\alpha }_{i}=0$ (${\forall }_{i} \le L-1$), where $L=\frac{p}{100}\times N$. Table 4 summarizes the number of layers trained for different setups.

**Table 4:** Details of different setups that are experimented

	Percentage of layers fine-tuned	Number of layers fine-tuned
Setup-1	10%	1
Setup-2	20%	3
Setup-3	30%	5
Setup-4	40%	7
Setup-5	50%	9
Setup-6	60%	11
Setup-7	70%	13
Setup-8	80%	15
Setup-9	90%	17
Setup-10	100%	19

To identify the best setup, we relied upon the 2 measures that include highest (or close to highest) validation accuracy and lowest (or close to lowest) validation loss. Best setups were identified independently for each of the three 3 CNNs that were designed to handle 3 magnification levels.

**Fig. 8:**
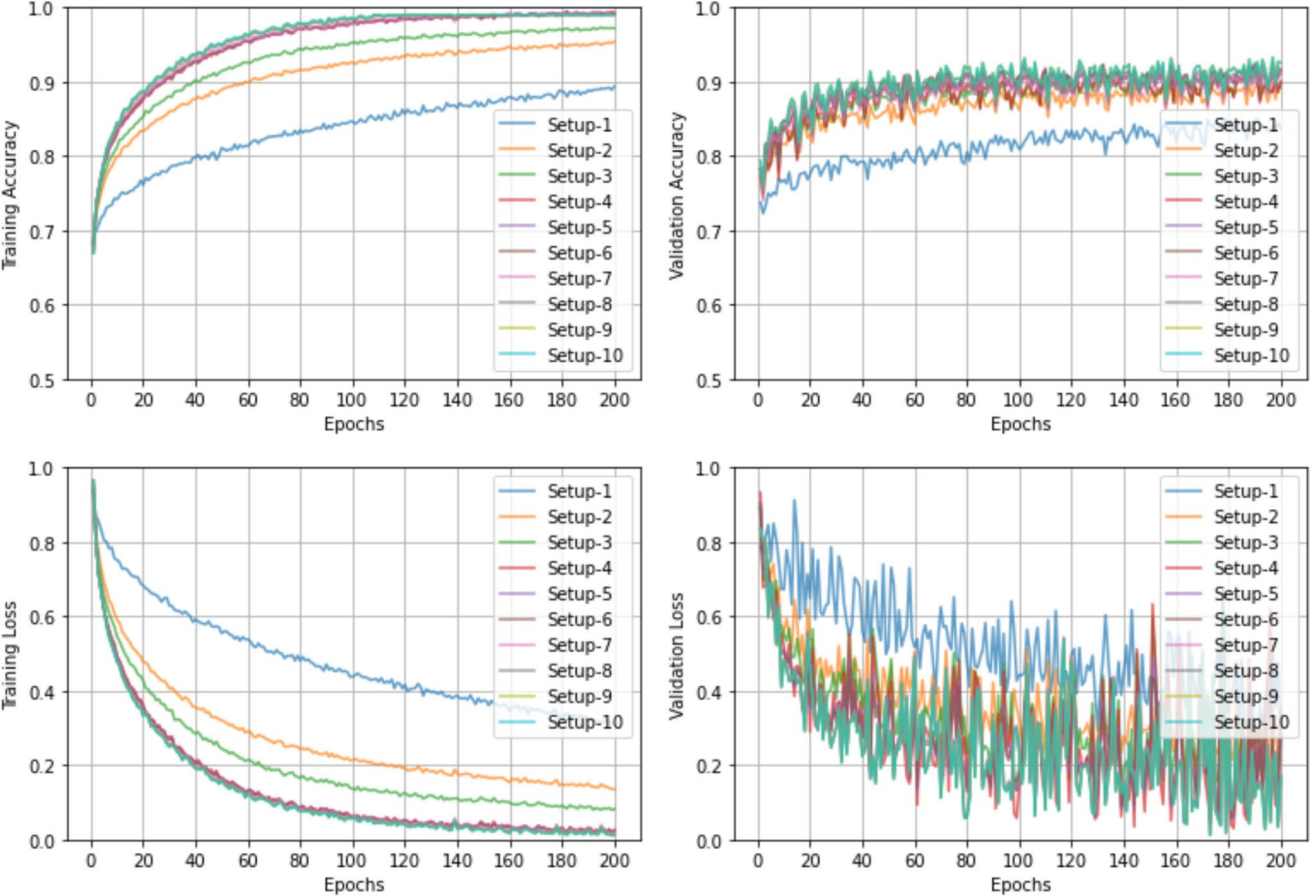
CNN training and validation for term-based classification. Training accuracy, validation accuracy, training loss and validation loss at the 5x magnification

Figure 8 shows the training accuracy, validation accuracy, training loss and validation loss at the 5x magnification.

From the accuracy plots, a steady increase in training accuracy over epochs is observable for all setups. Excluding setups 1, 2, and 3, all other setups exhibited very similar performance, reaching their saturation around 120 epochs. The validation accuracy also improved, but at a slower pace, stabilizing around 92% by epoch 120. In comparison to training accuracy, the validation accuracy curve demonstrates a zigzagging pattern.

From the loss plots, a steady decrease in training loss is also observable, with a very similar decreasing trend by setups 4–10. Unsurprisingly, the validation loss demonstrated some zigzagging patterns for all setups, which could be due to data variability, and training dynamics. However, an overall decreasing trend over the epochs is evident. Once again, setups 4–10 demonstrated comparable performance. Among the tested setups, setup 7 demonstrated the most balanced performance.

Table 5 summarizes the sensitivity, specificity, and accuracy of the CNNs at 5x, 10x and 20x magnifications.

**Table 5:** Sensitivity, specificity, and accuracy of CNNs for term-based classification on validation set

	5x [95% CI]	10x [95% CI]	20x [95% CI]
Sensitivity	92.61% [88.69%, 96.53%]	90.49% [86.57%, 94.41%]	83.95% [80.03%, 87.87%]
Specificity	99.25% [97.29%, 100%]	99.07% [93.19%, 100%]	90.13% [86.21%, 94.05%]
Accuracy	92.80% [88.88%, 96.72%]	91.04% [85.16%, 96.92%]	84.71% [82.75%, 86.67%]

The results in Table 5 indicate that the CNN trained at 5x magnification outperforms those trained at 10x and 20x magnifications across all evaluated metrics. The CNN trained at 10x magnification shows the second-best performance, followed by the CNN trained at 20x magnification. The superior performance at 5x magnification is likely due to the larger tissue area visible, providing more contextual information, which aids in better identifying patterns and structures characteristic of different tissue types.

**Fig. 9:**
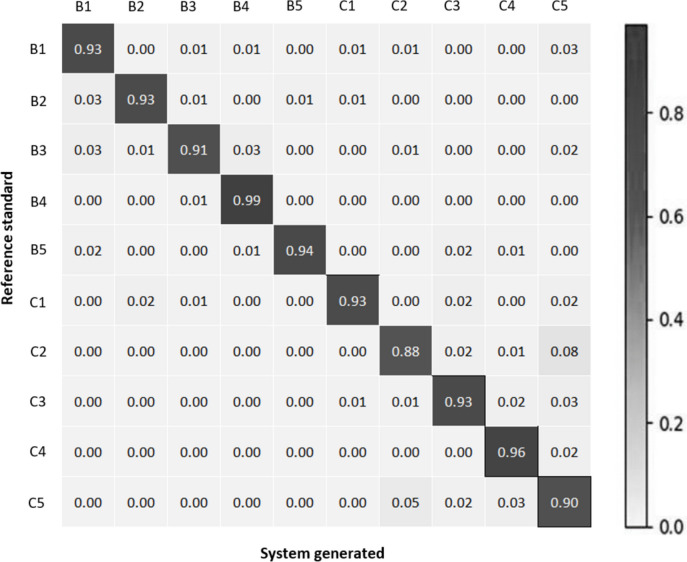

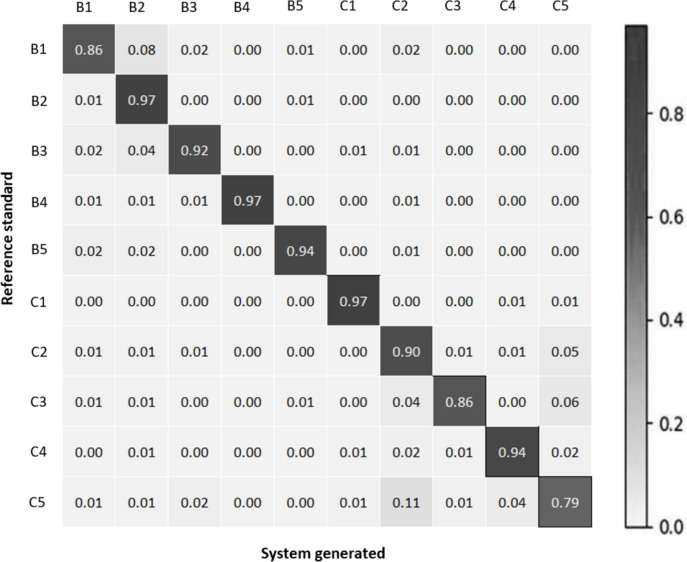

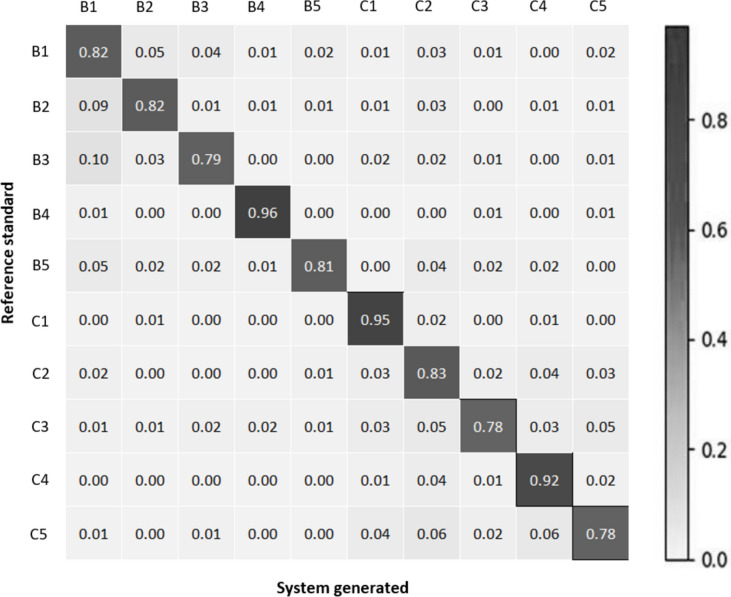
Normalized confusion matrix between reference standard and system generated output for term-based classification at **a** 5x magnification, **b** 10x magnification, and **c** 20x magnification

Figure 9 shows the normalized confusion matrix of the three CNNs on the validation set.

(a).

Figure 9 shows that while some misclassifications among terms are evident, cancerous terms tend to be misclassified as other cancerous terms, and the same trend is observed for non-cancerous terms. This observation is supported by the findings in Table 6, which presents increased performance metrics compared to Table 5. In addition with the findings on validation set, Table 6 also summarizes the sensitivity, specificity, and accuracy of the cancer versus benign classification task on the test set.

**Table 6:** Sensitivity, specificity, and accuracy of CNNs on cancer versus benign classification task

	Validation set			Test set		
	5x (%)	10x (%)	20x (%)	5x (%)	10x (%)	20x (%)
Sensitivity	98.20	97.40	97.00	93.46	93.14	88.23
Specificity	97.20	98.40	93.60	88.24	87.13	83.15
Accuracy	97.70	97.90	95.50	90.53	90.10	85.53

From the results in Table 6, it is apparent that the CNN trained at 5x magnification level demonstrates the best balance and robustness across both validation and test sets, showing superior generalization performance. Although the CNN trained at 10x magnification exhibits slightly better specificity and accuracy compared to the CNN trained at 5x magnification in the validation set, the 5x magnification model shows higher sensitivity (98.20%), indicating a strong ability to correctly identify positive cases, which is crucial. Furthermore, the CNN trained at 5x magnification outperforms the 10x magnification model in the test set across all measures. The CNN trained at 20x magnification level shows the weakest performance, especially on the test set, indicating that lower magnification (5x) appears to be more advantageous for achieving better performance in tissue analysis tasks.

### Reported Performance of Methods in Tissue Analysis

Table 7 summarizes the performance of some of the best reported methods in the context. It is important to emphasize that these findings based on different test datasets. Unsurprisingly, variability in test datasets can introduce significant differences in performance outcomes, making direct comparisons between methods difficult. We also found a great diversity of evaluation metrics used for performance evaluation in the literature, with most common being sensitivity, specificity, and accuracy. Therefore, the experimental results presented in this table should be viewed as a summary of findings reported in the literature rather than as direct comparisons. It is also worth mentioning that majority of these methods are limited to tissue classification only and, in different to ours, do not perform cancer portion analysis to meet clinical reporting needs.

**Table 7:** Performance analysis of different state-of-the-art methods in WSI-based prostate cancer diagnosis

Methods	Cancer vs benign	Gleason grading
[42]	AUC^a^: 0.85, sensitivity: 91.3%, specificity: 84%	Accuracy: 79%, sensitivity: 75.9%, specificity: 77.9%
[43]	Accuracy: 92%, sensitivity: 90%, specificity: 93%	Accuracy: 90%, sensitivity: 77%, specificity: 94%
[2]	AUC: 0.994, sensitivity: 99.03%, specificity: 93.74%	AUC: 95.63, sensitivity: 85.95%, specificity: 90.66%
[22]	Accuracy: 88.33%, recall (sensitivity): 91.92%	Accuracy: 93.16%, recall (sensitivity): 90.33%
[23]	Operating point 1: AUC: 0.9877, sensitivity: 98%, specificity: 90.2% Operating point 2: AUC: 0.9877, sensitivity: 99.1%, specificity: 73.6%	Accuracy: 73%
[24]	With ATARI: Accuracy: 89%, sensitivity: 81%, specificity: 81%	With ResNet101: Accuracy: 79%

^a^AUC=Area Under the Curve

Table 7 presents only the quantitative findings. For concise presentation, when multiple datasets were used for an experiment, their overall performance (i.e., the average) is computed and reported. When multiple models were proposed or evaluated in a single study, only the performance of the best-performing model is listed. For example, for Duenweg et al*.*’s study, we only reported the performance of the ATARI model for the cancer versus benign classification task, and the ResNet model for Gleason grading. When a model has been evaluated at multiple operating points with similar performance, we independently reported the performance of each. For example, Xiang et al*.*’s study, which evaluated model performance considering two operating points for the cancer versus benign classification task, is reported in this manner.

Based on Table 7, Pantanowitz et al*.* seems to achieve the highest sensitivity and specificity for both cancer versus benign classification and Gleason grading, with respective values of 99.03% and 93.74% for the former, and 85.95% and 90.66% for the latter. Lucas et al*.* seems to achieve the second-best overall performance, with sensitivity and specificity of 90% and 93% for cancer versus benign classification, and 77% and 94% for Gleason grading.

## Limitations of this Study

Each WSI was divided into multiple patches of size $256\times 256$; which were then used to train the models. Due to technical limitations, each annotated polygon that outlined the individual tissue term was cropped into a square region, and this square region was then further divided into multiple patches of size $256\times 256$. Unsurprisingly each square region contained some unannotated tissue regions, because of the cropping to a square region. Unannotated tissue regions are not a serious concern as long as they do not belong to any of the terms of interest. However, it could be a bottleneck in robustly training the model if it contains other terms of interest than the ones classified. Thus, each individual patch should be reviewed by experts and/or a more advanced patch generation technique needs to be developed.

In this work we limited ourselves to compare the classification performance of the models with human experts at patch levels. In future it would be interesting to analyse the slide level measures such as primary tumour pattern area size (in mm^2^), secondary tumour pattern area size (in mm^2^), minor tumour pattern area size (in mm^2^) etc. returned by the system with that of human experts.

In this work our focus was predominantly limited to classification of 10 terms only. In future it would be interesting to include more terms. At the same time, it would be interesting to aim for segmentation of those terms so that slide level tissue outlining of individual terms could be done precisely and measures such as area sizes could be computed more accurately.

A broad scale experiment on a larger dataset of WSIs collected from different labs and also representing diversity in Hematoxylin and eosin staining is necessary to further guide the developments.

## Discussion and Conclusions

In this study, we sought to develop an artificial intelligence system for automated assessment of prostate cancer using WSIs. A total of 2,340 WSI were used in this study, which include a training and validation dataset of 1,904 WSI and a separate hold-out (test) dataset of 436 WSI. We planned to use at least 10% of the whole dataset for testing. However, we managed to collect additional data (9% more) during the system development phase and have used them all in the testing. We compared the performance of the system using WSIs captured at three different magnification levels. We achieved the best performance of the system using WSI at 5x magnification. For term-based classification we obtained an overall (averaged over all terms) validation accuracy of 92.80% with sensitivity and specificity respectively 92.61% and 99.25%, when 5x magnification level is used. For 10x magnification, these performance metrics were respectively 91.04%, 90.49%, and 99.07%, and at 20x magnification they were 84.71%, 83.95%, 90.13%. For the cancer versus benign classification task, we obtained an accuracy of 97.70% on the validation set and accuracy of 90.53% on the test set, when 5x magnification was used. For the 10x and 20x magnification we found an accuracy of 97.90% and 95.50% on the validation set and 90.10% and 85.53% on the test set. It is worth mentioning that comprehensive annotations for all 10 terms were not available for the test set. Instead, we had clustered annotations categorizing these terms as either cancerous or benign. Therefore, only the performance of the cancer versus benign classification task was evaluated and reported for the test set. While the system allows to ensemble the 5x, 10x and 20x models, we haven’t observed any difference in the performance by ensembling them that would be relevant in practice. We also developed a traditional rule-based tissue detection method for initial assessment of tissue or background patches prior to sending the patches for CNN-based classification. Tissue detection was performed exclusively at the 5x magnification level. The information obtained at 5x magnification was subsequently used for the 10x and 20x magnification levels. This approach was adopted because performing tissue detection at the 10x level would increase the computational cost by four times, and at the 20x level, by sixteen times. Notably, tissue detection at the 5x magnification level has already been found to be 99.5% accurate.

There are no publicly available datasets specifically designed for evaluating different methods in this context. Additionally, we were unable to find codes for the best-performing methods that we could run on our datasets. Which has made direct comparisons between these methods particularly difficult. Consequently, we need to rely on the results and methodological descriptions reported in the literature. Compared to the system by [2], which is one of the best-reported methods in the literature, our system is simpler; however, it demonstrates comparable performance.. Further to that the system computes slide level measures such as primary tumour pattern area (in mm^2^), secondary tumour pattern area (in mm^2^), minor tumour pattern area (in mm^2^) etc., along with perineural invasion to meet clinical reporting needs.

Given the increasing burden of cancer on the healthcare system, the proposed automated system is highly likely to perform a vital role in decision support systems for diagnosis of prostate cancer. With the growing and critical role of whole slide imaging in the diagnosis of cancer, the proposed automated system should be of clinical value, not only for increasing diagnostic accuracy and efficiency in clinical practice, but also in reducing the healthcare cost significantly.

## Figures and Tables

**Fig. 10:**
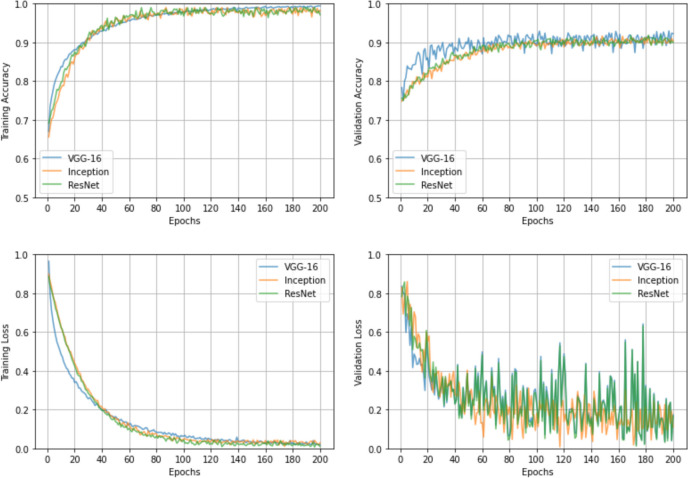
Training accuracy, validation accuracy, training loss and validation loss by different CNNs at the 5x magnification

## Data Availability

The dataset used in this study is private and is not publicly available at this time. Access to the data (or partial data) may be granted upon reasonable request, subject to approval by the data owners and relevant ethical considerations.

## Appendix

Fig. 10.

## Experimental Evaluation of Deep Learning Architectures for Tissue Classification

To comprehensively assess the performance of various deep learning architectures for tissue classification, we conducted experiments using VGG-16, Inception-v3, and ResNet. Each model was trained on the same dataset to ensure a fair comparison of their capabilities. VGG-16, renowned for its depth and simplicity, served as a robust baseline with a straightforward yet powerful convolutional architecture. Inception-v3, characterized by its intricate design and inception modules, aimed to capture a broader range of features through multi-scale convolutions. ResNet, utilizing residual learning and skip connections, was employed to mitigate the vanishing gradient problem, enabling effective training of deeper networks. Throughout the experiments, we monitored two key performance metrics: training and validation accuracy, and training and validation loss. We explored various hyperparameter configurations, starting with the default or commonly used settings. Figure A-1 illustrates the training accuracy, validation accuracy, training loss, and validation loss curves obtained for the optimal setups of these CNNs.
